# Stereoselective deprotonation installs an unusual *Z*-8,9 double bond during biosynthesis of the diterpene pheromone sobralene†‡

**DOI:** 10.1039/d5cc03298a

**Published:** 2025-07-07

**Authors:** Igor F. P. Da Silva, Charles Ducker, John A. Pickett, Antônio E. G. Santana, Neil J. Oldham

**Affiliations:** a School of Chemistry, University of Nottingham, University Park Nottingham NG7 2RD UK neil.oldham@nottingham.ac.uk; b Institute of Chemistry and Biotechnology, Federal University of Alagoas A. C. Simões Campus Maceió AL 57072-970 Brazil; c Agricultural Sciences Centre, Federal University of Alagoas Rio Largo AL 57100-000 Brazil; d School of Chemistry, Cardiff University Cardiff CF10 3AT UK

## Abstract

A terpene synthase from the sandfly *Lutzomyia longipalpis*, catalyses stereoselective removal of the *pro*-S proton from C8 of geranylgeranyl diphosphate to generate the characteristic (*Z*)-8,9 double bond seen in the diterpene pheromone sobralene. Retention of deuterium in structurally related minor products demonstrates that they are not produced *via* sobralene, but are made directly by the enzyme.

## Abstract 2

Biosynthesis of the novel diterpene sobralene proceeds by elimination of the *pro*-S proton from C9 of its precursor carbocation, catalysed by the insect terpene synthase *Ll*TPS1.

## Graphical abstract

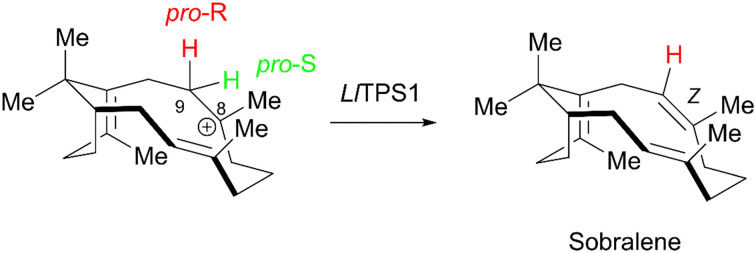

Terpenes represent a highly diverse group of natural products that often possess pronounced biological activity.^1^ Amongst eukaryotes, plants and fungi are recognized as the major producers, but insects also employ terpenes (and terpenoids) for a range of ecological functions, such as pheromone-based communication and defence.^2,3^ In insects, the mevalonate (MVA) pathway appears to be the exclusive route to the isoprenoid building blocks of terpenes, namely dimethylallyl diphosphate (DMAPP) and isopentenyl diphosphate (IPP), which can then be assembled into longer isoprenyl diphosphate chains by the action of isoprenyl diphosphate synthases (IDSs).^2^ Whilst this pathway has long been studied, it is only recently that knowledge of insect terpene synthases (TPSs) has emerged.^4–6^ First to be discovered was the so-called non-canonical class of insect TPSs that do not resemble the more familiar TPSs seen in plants and microorganisms but, instead, are derived directly from IDSs. These enzymes have lost their ability to recruit IPP in order to extend the isoprenyl chain and hence perform only the first (TPS-like) step of their mechanism, which is to generate a reactive carbocation intermediate by catalysing the loss of inorganic diphosphate (PPi) from isoprenyl diphosphate substrate.^7^ Following the identification of several examples of this class of TPS, a natural tendency has existed to assume that all insect TPSs were IDS-derived. Very recently, however, it has been shown that some insects, specifically of the Sciaridae family, possess canonical TPSs that are similar to those seen in plants and microorganisms.^8^ Thus, the picture of terpene biosynthesis in insects is both interesting and complex.

Sobralene (1) (Fig. 1) is a novel male-produced diterpene (C_20_) sex/aggregation pheromone used by populations of the sandfly, *Lutzomyia longipalpis*.^9^ In Central and South America, this blood-feeding insect is a major vector of the *Leishmania* parasite, which causes the neglected tropical disease leishmaniasis.^10–12^ Sobralene is a diterpene with a 6,12-membered ring-fused structure related to the verticillenes but differs due to the presence of an unusual (*Z*)-8,9 double bond. This contrasts with the (*E*)-7,8 double bond seen in verticillene (2), which reflects that in the equivalent position of the geranylgeranyl diphosphate (GGPP) precursor (Scheme 1).^13^ Diterpene 2 is a minor product of the *L. longipalpis* sobralene synthase *Ll*TPS1 as are cembrene A (3) and phomactatriene (4).^14^ Product stereochemistry is currently unknown.

**Fig. 1:**
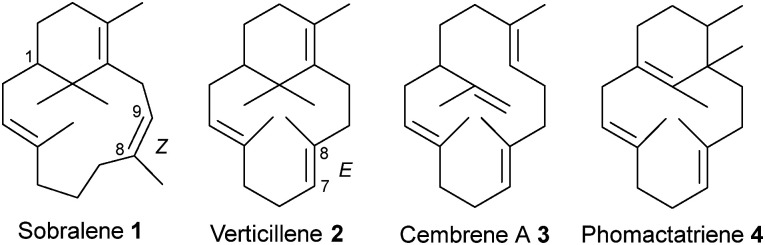
Structures of sobralene (1) and the minor diterpene products of *Ll*TPS1.

**Scheme 1:**
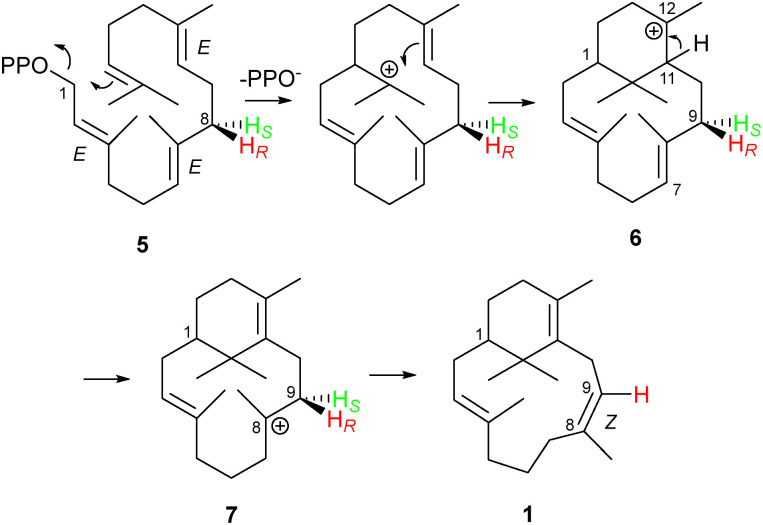
Proposed biosynthetic route to sobralene (1) from GGPP (5) *via* the C12- and C8- and cations (6) and (7), respectively, showing loss of one of the C9 protons from 7. Transformation of 6 to 7 likely proceeds through two 1,5-proton shifts *via* a C4 cation.

To facilitate formation of the (*Z*)-8,9 double bond in sobralene, a double bond migration is required with an accompanying proton loss from the intermediate carbocation. Here we show that, when catalysed by *Ll*TPS1, this step proceeds by stereoselective elimination of the *pro*-S proton from C8 of GGPP. Moreover, we demonstrate (i) retention of deuterium in verticillene (2), cembrene A (3) and phomactatriene (4), revealing that they are derived directly from GGPP and not produced *via* sobralene (1), and (ii) that the *L. longipalpis* GGPP synthase (GGPPS) catalyses addition at the *Si*-face of IPP, as seen with FPP synthases (FPPSs).

During the biosynthesis of sobralene (1) from GGPP (5) two ring closure steps are required to generate the C12-verticillenyl cation intermediate (6) (Scheme 1). These steps are presumably followed by proton transfer from C11 to C7, an action which both generates the C11,12 double bond and creates a C8-carbocation (7). By reference to taxadiene biosynthesis, Palframan *et al.* have postulated that proton transfer proceeds in two steps *via* C3,^9^ where a double 1,5-proton shift has been postulated and evidenced in this system.^15–18^ Once formed, the C8-verticillenyl cation (7) can be converted to sobralene (1) by loss of a proton from C9, which forms the (*Z*)-8,9 double bond.

To identify the stereochemistry of proton elimination from the C8-verticillenyl cation (7) we utilised enantiotopically labelled (8*S*)-(4,8-^2^H_2_)-GGPP (10). Due to a change in atom numbering following ring closure, C8 in GGPP becomes C9 in the verticillenyl cation and, ultimately, in sobralene (Scheme 1). Hence, this diphosphate precursor can report if either the *pro*-R or *pro*-S proton on C9 is lost in the formation of sobralene's (*Z*)-8,9 double bond. Inspired by the work of Cane,^19^ (8*S*)-(4,8-^2^H_2_)-GGPP (10) was accessible from incubation of GPP (8, *n* = 2) and (*E*)-(4-^2^H_1_)-IPP (9) with the FPPS from *L. longipalpis* (*Ll*FPPS), since (i) this enzyme produced the over-extended GGPP product, as well as FPP, and (ii) the stereochemistry of FPPSs is well established due to the work of Cornforth and Popják (see Fig. S1 for structural identity, ESI‡).^20^ Thus, elongation of GPP with (9) resulted in (4*S*)-(4-^2^H_2_)-FPP and ultimately in (8*S*)-(4,8-^2^H_2_)-GGPP (10). Note: although the stereochemistry at C4 in GGPP was also likely to be (*S*)-, given that this stereocentre arises from a non-standard elongation by an FPPS, we did not assume that it followed the established convention. Importantly, however, stereochemistry at C8 in GGPP – the position of interest – was well defined by the normal action of an FPPS. (*E*)-(4-^2^H_1_)-IPP (9) was synthesised using the approach of Ito *et al.*^21^

Incubation of GPP and (*E*)-(4-^2^H_1_)-IPP (9) with *Ll*FPPS and the sobralene synthase *Ll*TPS1 produced sobralene (13) (Scheme 2) with only a single deuterium atom, as determined by GC-MS analysis (Fig. S2, ESI‡). Cembrene A, verticillene and phomactatriene, however, all possessed two deuterium atoms (Fig. S3–S5, ESI‡). To confirm that deuterium loss in sobralene occurred at C8, and not C4 of (8*S*)-(4,8-^2^H_2_)-GGPP (10), FPP (8, *n* = 3) was elongated with a single unit 9 to install a deuterium atom at C4 of GGPP only (11) (Scheme 2). Following co-incubation with *Ll*TPS1 and GC-MS analysis, (^2^H_1_)-sobralene (13) was found exclusively, which possessed an identical EI-MS spectrum to that seen in the previous experiment, and confirmed retention of the C4 deuterium atom from GGPP (Fig. S2, ESI‡). Cembrene A, verticillene and phomactatriene were also all labelled with a single deuterium atom, as expected (Fig. S3–S5, ESI‡). Taken together, these results demonstrate that the C8 deuterium atom from GGPP, which becomes C9 in the verticillenyl cation is lost during double bond migration leading to sobralene. Hence, it is the *pro*-S proton that is eliminated from cation 7. Moreover, the retention of both deuterium atoms from (8*S*)-(4,8-^2^H_2_)-GGPP in cembrene A, verticillene and phomactatriene showed that these diterpenes were direct products of *Ll*TPS1 and could not have been produced *via* sobralene. Palframan *et al.* have previously shown that sobralene could be converted to verticillene under acid catalysed conditions,^22^ but it was clear from the labelling results presented here that this was not the origin of verticillene, or the other diterpenes.

**Scheme 2:**
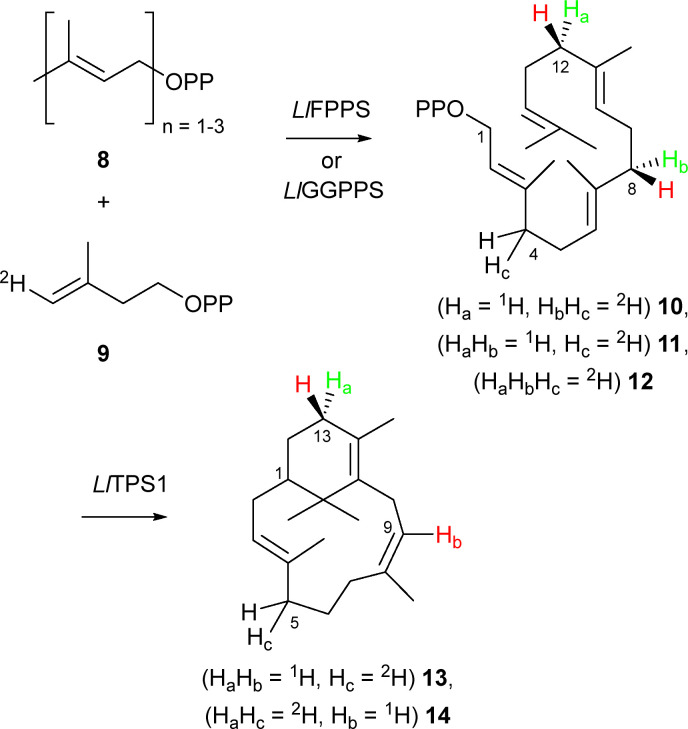
Production of GGPP isotopologues 10–12 by *Ll*FPPS and *Ll*GGPPS and their conversion to sobralene through elimination of ^2^H from C8 of GGPP = C9 sobralene.

To complete the set of experiments with *Ll*FPPS and *Ll*TPS1, we next elongated DMAPP (8, *n* = 1) with (*E*)-(4-^2^H_1_)-IPP (9) to install three deuterium atoms in GGPP, *i.e.* (8*S*,12*S*)-(4,8,12-^2^H_3_)-GGPP (12) (Scheme 2). This led to sobralene possessing two deuterium atoms (14), with the other diterpenes retaining all three (Fig. S2–S5, ESI‡). The production of isotopologues of the diterpene products of *Ll*TPS1, which possessed deuterium atoms at well-defined positions (following established biosynthetic rules), provided useful insights into the MS fragmentation of these compounds (Fig. S9, ESI‡).

Following successful production of deuterium labelled GGPPs using *Ll*FPPS, and their conversion into the diterpene products of *Ll*TPS1, we next explored *Ll*GGPPS^23^ as an IDS in the coupled reaction. Although GGPPSs are the natural enzymes for producing diterpene precursors, their stereochemistry is less well established than that of FPPSs, hence our use of the latter in producing (8*S*)-(4,8-^2^H_2_)-GGPP (10) thus far. Incubation of GPP and (*E*)-(4-^2^H_1_)-IPP (9) with *Ll*GGPPS gave an identical set of results to those obtained with *Ll*FPPS, namely sobralene possessed a single deuterium label (13), whilst the other diterpene products were doubly labelled. Thus, *Ll*GGPPS must also incorporate the (*E*)-H atom from IPP into the *pro*-S position on C4 in the elongated isoprenyl diphosphate through *Si*-face addition to IPP.

In an insightful computational study of the likely intermediates involved in sobralene biosynthesis, Hayes *et al.* used product 1 as a starting point for the cation conformation (7a) (Scheme 3) needed to install the (*Z*)-8,9 double bond.^24^ From this they predicted that the *pro*-S proton on C9 would be eliminated. Interestingly, however, this conformation was found to be slightly higher in energy (2.3 kcal mol^−1^) than 7b, which is required to produce verticillene (2). Our experimental finding here that the *pro*-S proton on C9 is indeed eliminated to generate 1 means that *Ll*TPS1 must, therefore, push the conformational equilibrium in favour of 7a. The presence of 2 as a direct (minor) product of *Ll*TPS1 shows both that 7b has an equilibrium presence in the active site and that deprotonation at C7 is also possible. Direct production of cembrene A (3) and phomactatriene (4) by *Ll*TPS1 demonstrates that deprotonation at other sites is catalysed by the enzyme.

**Scheme 3:**
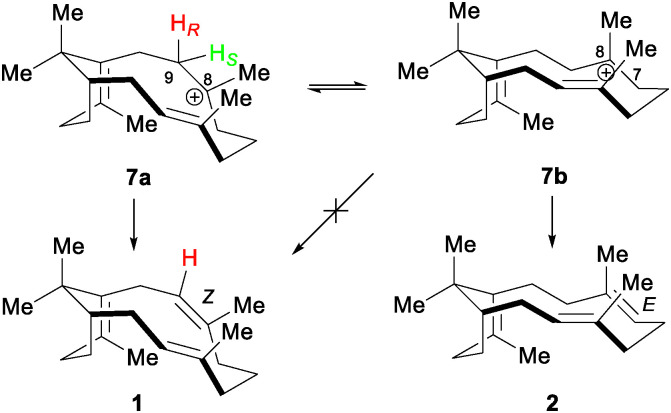
Cation conformation 7a required to produce sobralene (1), by elimination of the *pro*-S proton (green) from C9, and alternative conformation 7b that gives rise to verticillene (2). After Hayes *et al*.

In summary, the experiments described here provide three key pieces of information on the biosynthesis of sobralene (1): (i) that the *pro*-S proton is eliminated from cation 7 and that, therefore, this cation must principally adopt conformation 7a in the active site of *Ll*TPS1, (ii) that the minor diterpene products of the enzyme 2-4 are not produced from rearrangement of 1, but are directly formed from their corresponding cation intermediates, and (iii) that the *L. longipalpis* GGPPS, *Ll*GGPPS, demonstrates the same *Si*-face addition to IPP as seen for FPPSs.

Conceptualization: NJO; supervision: NJO, AEGS, JAP; investigation: IFPD, CD; writing: NJO, CD, IFPD, AEGS, JAP; funding acquisition: NJO, JAP.

This work was supported by the Biotechnology and Biological Sciences Research Council (BBSRC): research grant BB/V003933/1 to NJO and JAP, and by CAPES: PhD Abroad Sandwich studentship to IFPDS.

## Conflicts of interest

There are no conflicts to declare.

## Supplementary Material

CC-061-D5CC03298A-s001

## Data Availability

Additional supporting data are provided in the ESI‡ and are openly available from the University of Nottingham data repository at DOI: https://doi.org/10.17639/nott.7563.
