# Supplementary material for: Stereoselective deprotonation installs an unusual Z-8,9 double bond during biosynthesis of the diterpene pheromone sobralene
Source: Chem Commun (Camb). 2025 Jul 7;61(64):12026–9. doi: 10.1039/d5cc03298a (PMC12246896; doi:10.1039/d5cc03298a)
Supplement: CC-061-D5CC03298A-s001 [file CC-061-D5CC03298A-s001.pdf]

Supplementary Information (SI) for Chemical Communications.
This journal is © The Royal Society of Chemistry 2025

Supporting Information for

Stereoselective deprotonation installs unusual *Z*-8,9 double bond during biosynthesis of the diterpene pheromone sobralene

Igor F. P. Da Silva,[a,b] Charles Ducker,[a] John A. Pickett[c], Antônio E. G. Santana,[b] and Neil J. Oldham[a]*

Materials and Methods

General

Purified water (18.2 MΩ) was produced using an ELGA Purelab system (ELGA, High Wycombe, UK). Organic solvents were sourced from Merck (Feltham, UK) unless otherwise stated. NMR spectroscopy was performed on a Bruker Avance III or a Bruker Avance III HD spectrometer (400 MHz ^{1}H, 101 MHz ^{13}C and 162 MHz ^{31}P, Bruker, Billerica, MA) with chemical shift δ quoted in ppm, relative to TMS (^{1}H and ^{13}C) or phosphoric acid (^{31}P), and coupling constant *J* quoted in Hz. High resolution electrospray ionisation mass spectrometry was performed on an LTQFT Ultra hybrid linear trap-FTICR MS (Thermo Scientific UK) operated in negative ion mode, with a resolving power of 100,000 at *m/z* 400.

Protein Expression and Purification

Proteins from *L. longipalpis* were expressed and purified as described previously.[1,2] Briefly, pET100/D-TOPO^{Tm} bacterial expression vector encoding either *Ll*FPPS (XP_055677521.1, aa 72-411), *Ll*GGPPS (XP_055688734.1) or *Ll*TPS1 (XP_055691875.1; GeneArt, Thermo Fisher Scientific, Loughborough, UK) were transformed into BL21(DE3)pLysS *E. coli*, and single colonies were used to inoculate 20 ml LB cultures (100 µg/ml ampicillin) and incubated overnight at 37 °C and 200 rpm). The following morning, these cultures were added to 1L LB (100 µg/ml ampicillin) and incubated at 37 °C and 200 rpm until reaching OD_{600} ~0.5, then induced with 0.75 mM IPTG. They were then incubated at either 37 °C for 3 hours (*Ll*FPPS and *Ll*GGPPS) or 30 °C for 4 hours (*Ll*TPS1), before harvesting by centrifugation (14000 rpm, 4 °C, 20 min) and storage at -80 °C.

Bacterial pellets were lysed in 30 ml wash buffer (50 mM Tris pH 7.4, 100 mM NaCl, 20 mM imidazole, 10% v/v glycerol, 1 mM DTT) supplemented with 1% v/v Igepal CA-630 and EDTA-free cOmplete protease inhibitor (Roche, Welwyn Garden City, UK) with sonication on ice (25 Hz, 80% output, 12 x 10 sec) followed by centrifugation (14000 rpm, 4 °C, 20 min). Nickel-NTA agarose beads (Qiagen, 0.5 ml bed) were added to the soluble fraction and incubated with rotation at 4 °C for 90 min, before washing with 3 x 10 ml wash buffer and elution with 3 ml elution buffer (wash buffer containing 400 mM imidazole). Eluted protein was buffer exchanged to protein storage buffer (25 mM MOPSO pH 7.2, 100 mM NaCl, 10 % v/v glycerol, 1 mM DTT) using PD-10 desalting columns (Cytiva, Little Chalfont, UK), and aliquots were snap frozen (LN_2) and stored at -80 °C.

Synthesis of (*E*)-(4-$^{2}H_{1}$)-IPP

Deuterium labelled IPP was synthesised from 3-methyl-3-buten-1-ol using a literature method[3] which utilised lithium-halogen exchange of (*E*)-4-bromo-3-methyl-3-buten-1-ol followed by quenching with D_2O, tosylation and conversion to the diphosphate to yield 122 mg of product. ^{1}H NMR (400 MHz, D_2O) δ 4.74 (s, 1H), 3.97 (q, 2H, *J* = 6.70 Hz), 2.31 (t, 2H, *J* = 6.64 Hz),

1.69 (s, 3H); ^{13}C NMR (101 MHz, D_2O) δ 143.81, 111.16 (t, $J_{C\text{-}D}$ = 23.54 Hz), 64.02 (d, $J_{C\text{-}P}$ = 5.58 Hz), 37.81 (d, $J_{C\text{-}P}$ = 7.58 Hz), 21.59; ^{31}P NMR (162 MHz, D_2O) δ -6.96 (d, $J_{P\text{-}P}$ = 21.82 Hz), -10.61 (d, $J_{P\text{-}P}$ = 21.82 Hz) (Fig. S6). HR-MS: found 246.0048 ($(M\text{-}H)^-$), calculated for $C_5H_{10}DO_7P_2^-$ = 246.0048 (Fig. S7).

Isoprenyl Diphosphate Synthase (IDS) – Terpene Synthase (TPS) Assays

Coupled IDS-TPS assays were performed as follows: *Ll*FPPS or *Ll*GGPPS (2 µM) was incubated with 100 µM allylic substrate (DMAPP, GPP or (*E,E*)-FPP) and stoichiometric concentrations of (*E*)-(4-$^2H_1$)-IPP for GGPP production (300 µM with DMAPP, 200 µM with GPP or 100 µM with (*E,E*)-FPP) in assay buffer (25 mM MOPSO pH 7.2, 10 mM $MgCl_2$). After 1 hr incubation at 30 °C, *Ll*TPS1 (2 µM) was added and the aqueous solution overlaid with 200 µl pentane and incubated at 30 °C for 16 hrs. The organic and aqueous layers were then mixed with a Pasteur pipette, followed by centrifugation (3000 xg, 2 min, 4 °C). The aqueous layer was discarded and the organic layer was evaporated to ~50 µl under a steam of nitrogen and submitted to GC-MS analysis.

To confirm successful production of isoprenyl diphosphate intermediates, instead of TPS addition, either shrimp alkaline phosphatase (20 U, New England Biolabs, Ipswich, USA) was added and the resulting alcohols were subjected to GC-MS analysis, or the IDS products were desalted using a ZipTip (Merck Millipore, Watford, UK) desalting and the isoprenyl diphosphates measured directly by negative ion ESI-MS (Fig. S8).

Gas Chromatography – Mass Spectrometry (GC-MS)

Samples were assessed by GC-MS using either an Agilent 7890B GC equipped with a DB-5ms column (30 m × 0.25 mm × 0.25 µm, Agilent, Cheadle, UK) coupled to a Jeol AccuTOF GCx system (Jeol, Tokyo, Japan), or a Thermo ISQ 7000 GC–MS system equipped with a Restek Rtx-1701 column (30 m × 0.25 mm × 0.25 µM, Thermo Fisher Scientific, Loughborough, UK). For the Agilent GC the oven temperature was held at 35 °C for 4 min, then increased at 10 °C/min to 300 °C followed by a 6-min hold. For the Thermo GC, the oven temperature was held at 35 °C for 3 min, followed by an increase of 10 °C/min to 260 °C and a 5-min hold. Both instruments were operated in splitless mode and the inlet temperature was 200 °C. For the Agilent GC the helium carrier gas was set at 1 ml/min and the sample load volume was 5 µl, while for the Thermo GC the carrier gas was set at 1.5 ml/min and the sample load volume was 2 µl. Both MS instruments were operated in positive electron ionisation mode, with the mass range scanned *m/z* 40-500 in the Jeol MS (8-min solvent delay) and *m/z* 40-450 in the Thermo GC (5-min solvent delay).

References

1. C. Ducker, S. French, M. Pathak, H. Taylor, A. Sainter, W. Askem, I. Dreveny, A.E.G. Santana, J. A. Pickett and N.J. Oldham, *Insect Biochem. Mol. Biol.,* 2023, **161**, 104001.

2. C. Ducker, C. Baines, J. Guy, A.E.G. Santana, J. A. Pickett and N.J. Oldham. *Proc. Natl. Acad. Sci. USA*, 2024, **121**, e2322453121.

3. M. Ito, M. Kobayashi, T. Koyama and K. Ogura, *Biochemistry*, 1987, **26**, 4745.

Results

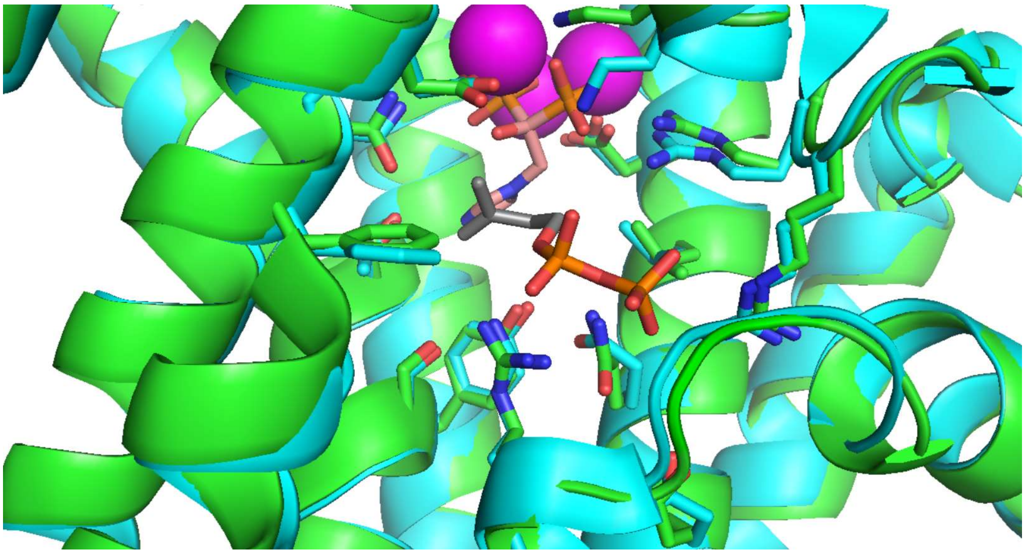

Figure S1. Aligned structures of an AlphaFold *Ll*FPPS model (green) and human FPPS (cyan, crystal structure PDB 2F8Z) showing near identical arrangement of key active site residues. Mg^{2+} ions (magenta spheres), zoldronate (pink, which occupies the DMAPP binding site), and IPP (grey), from the 2F8Z structure are also shown. The *si* face of IPP is facing towards zoldronate (with the former's Me group pointing up and the double bond to the left).

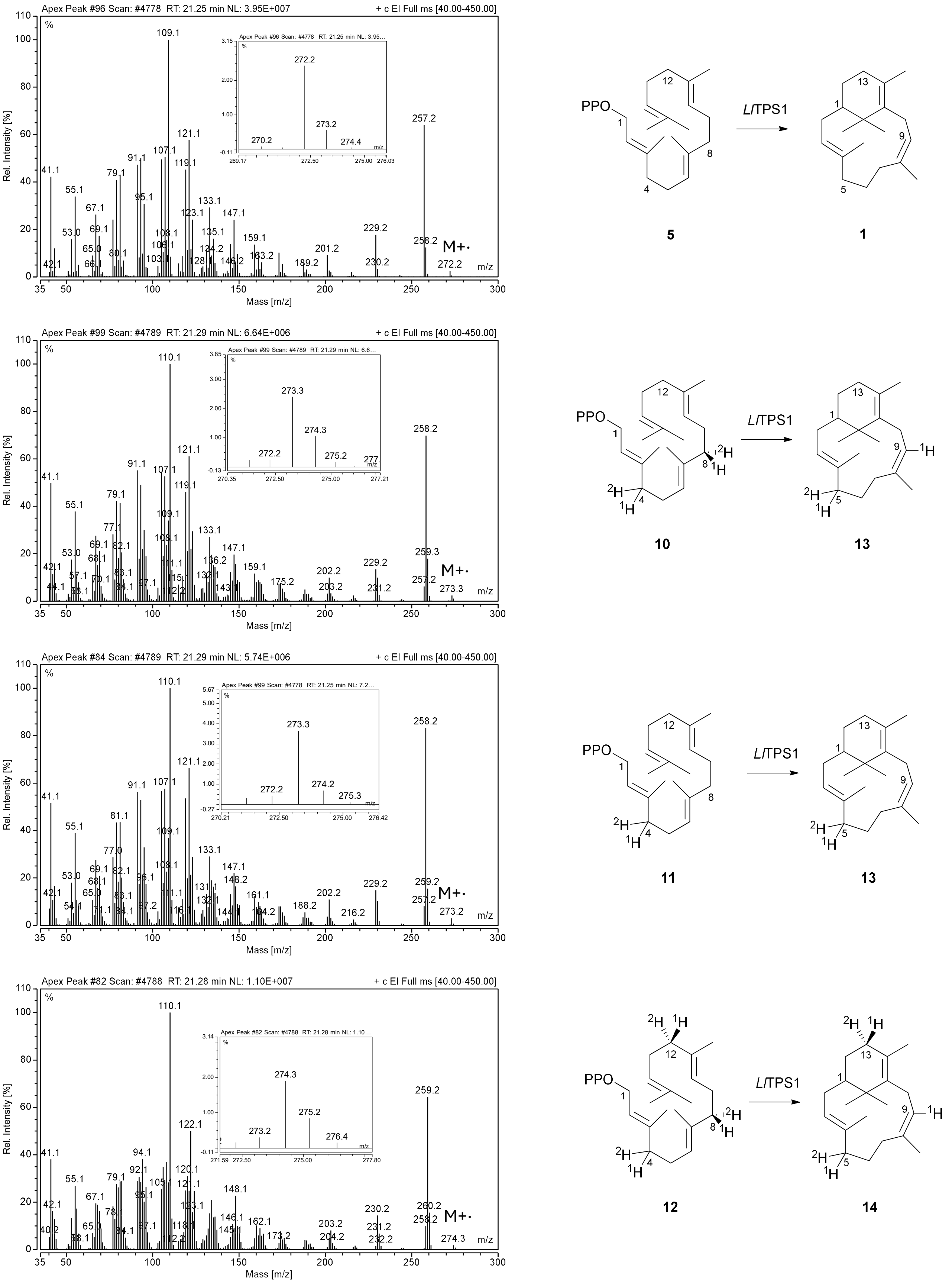

Figure S2. EI mass spectra of sobralene produced from isotopologues of GGPP indicated.

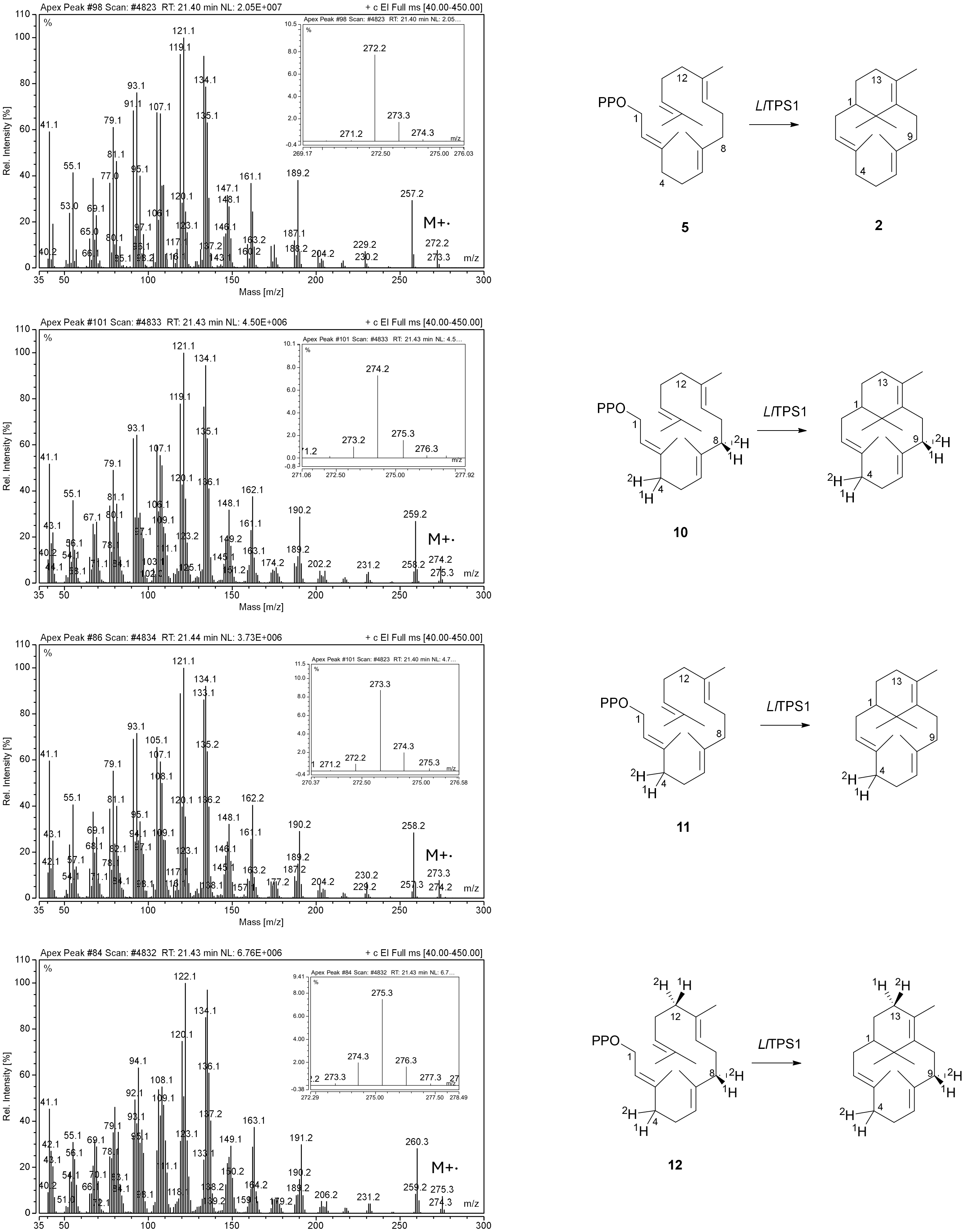

Figure S3. EI mass spectra of verticillene produced from isotopologues of GGPP indicated.

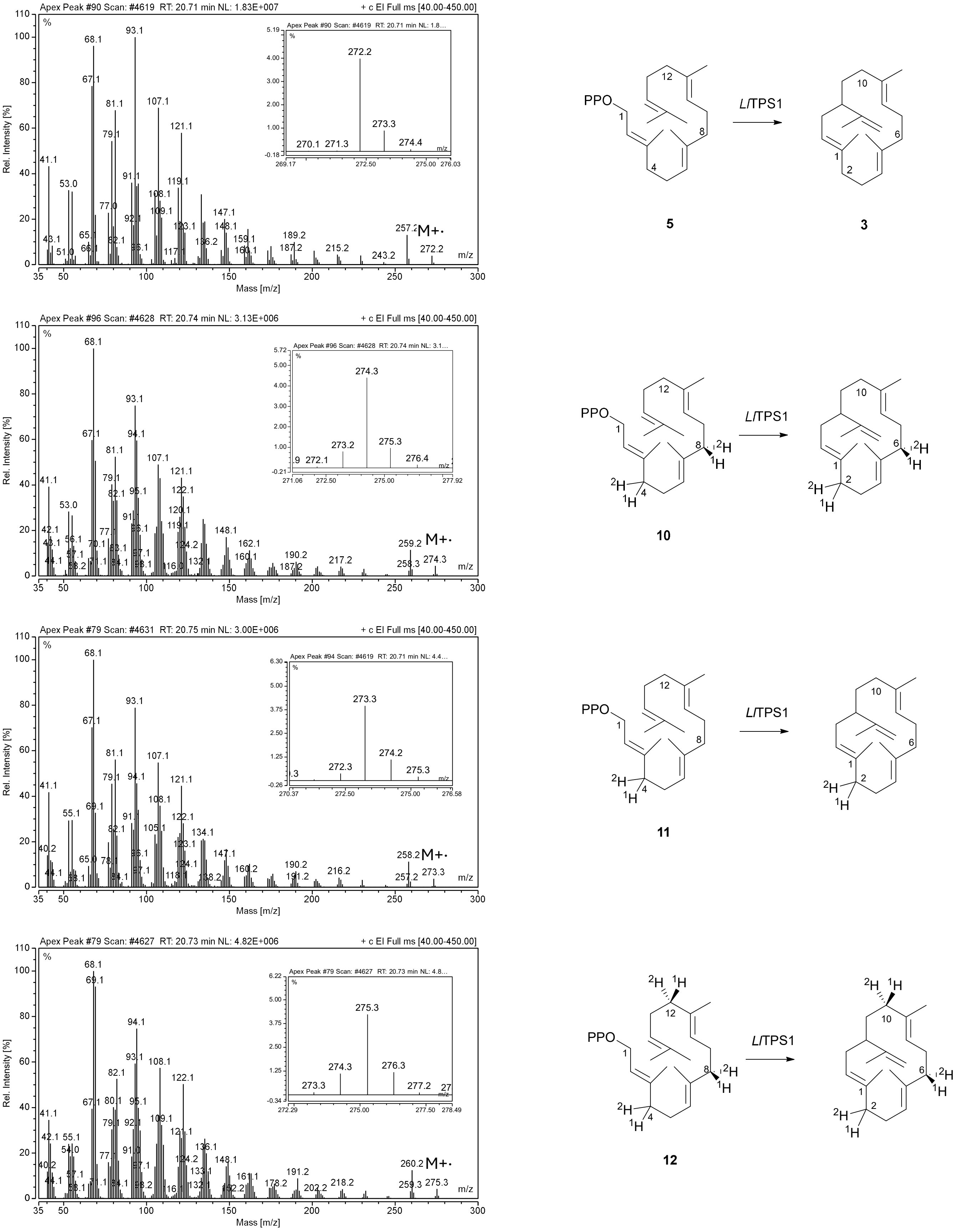

Figure S4. EI mass spectra of cembrene A produced from isotopologues of GGPP indicated.

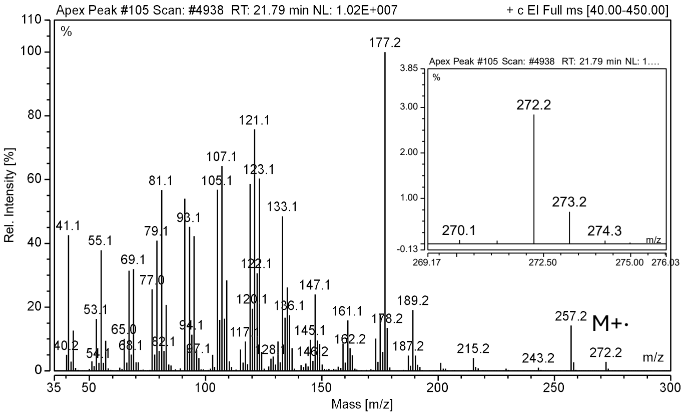

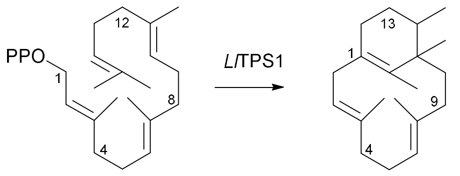

5

4

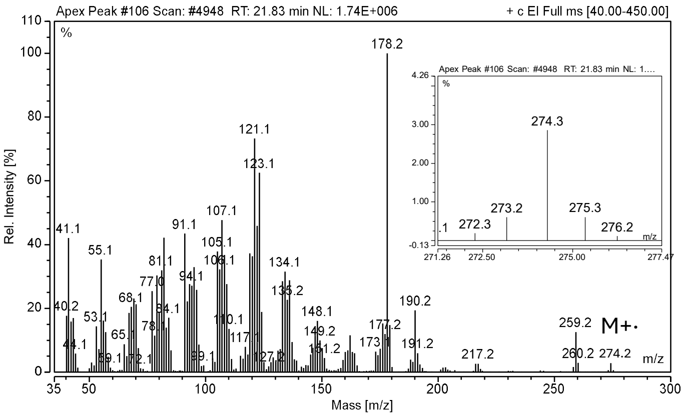

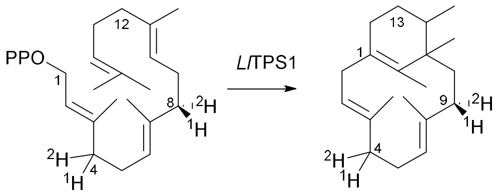

10

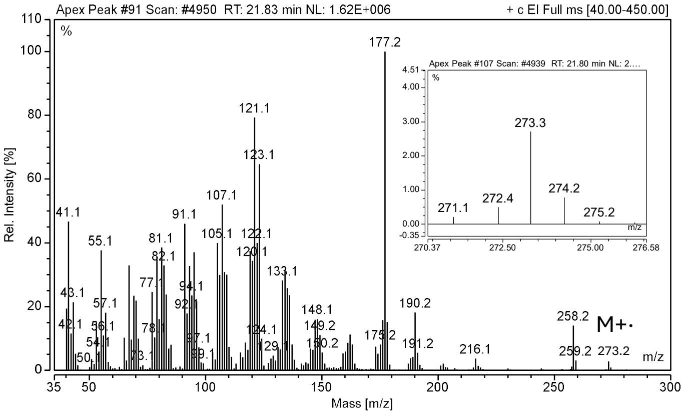

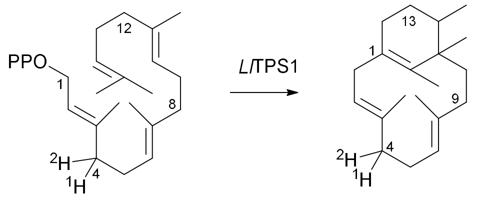

11

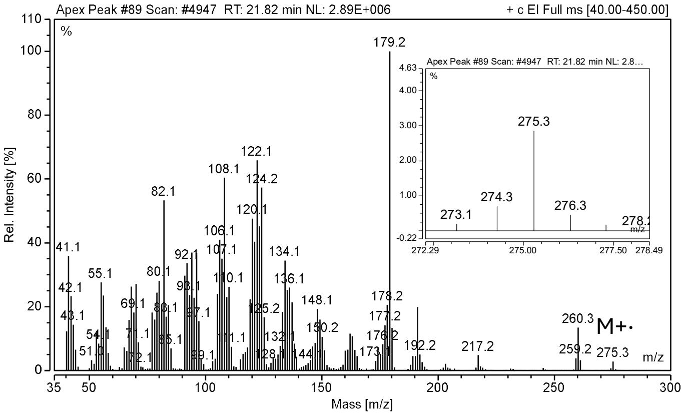

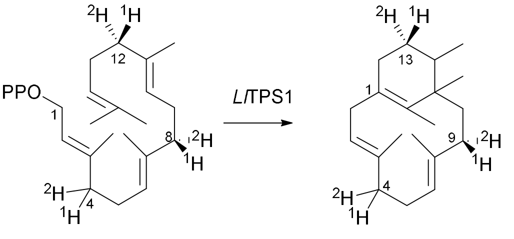

12

Figure S5. EI mass spectra of phomactatriene produced from isotopologues of GGPP indicated.

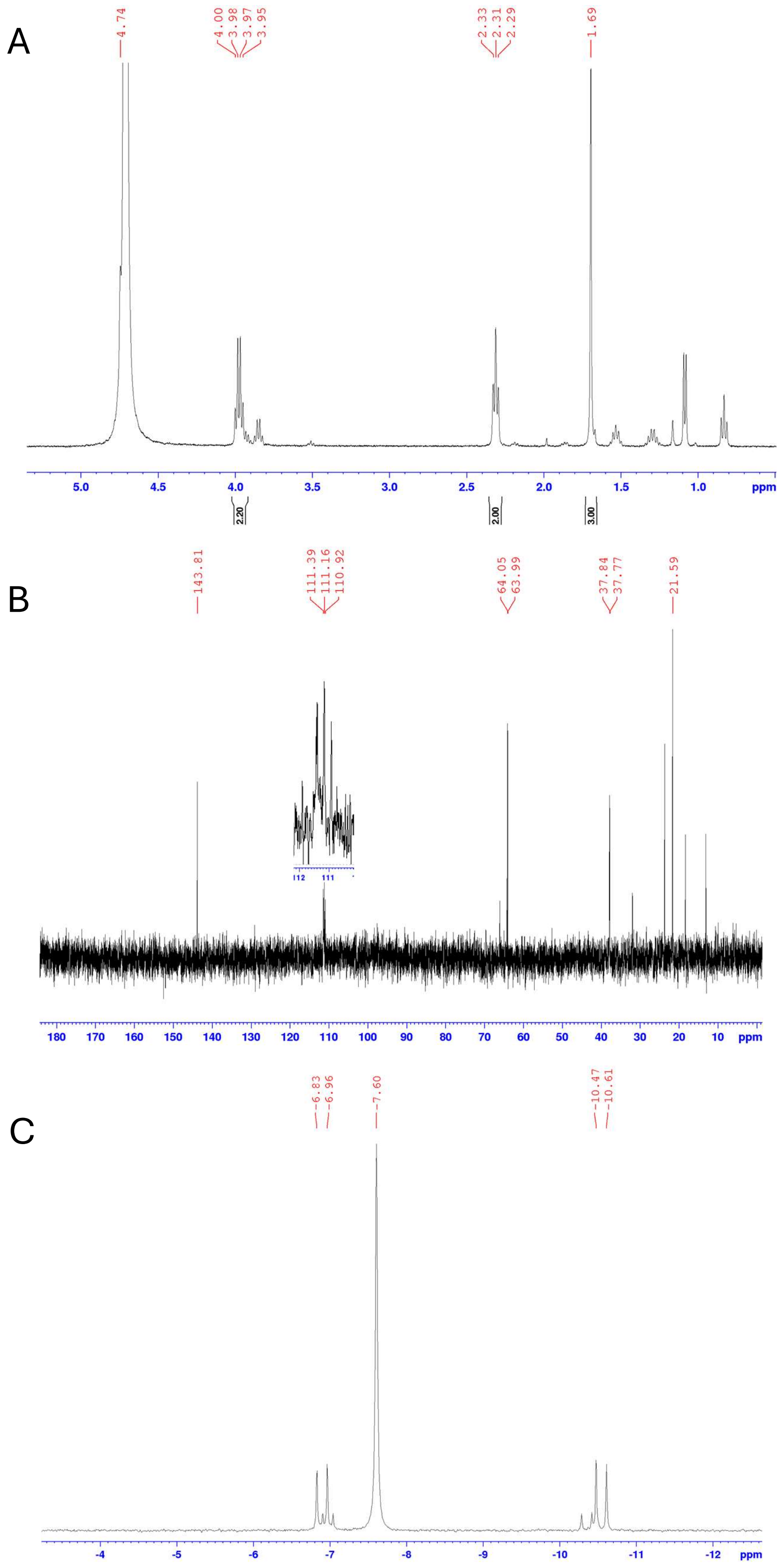

Figure S6. (A) ^{1}H, (B) ^{13}C NMR and (C) ^{31}P NMR spectra of (*E*)-(4-$^{2}H_{1}$)-IPP. Signals due to minor contaminants (likely *n*-BuPP, from the Li-halogen exchange step and isopropanol) can be seen in the spectra. We could not remove the former by column chromatography, but it did not interfere with the enzyme catalysed reaction.

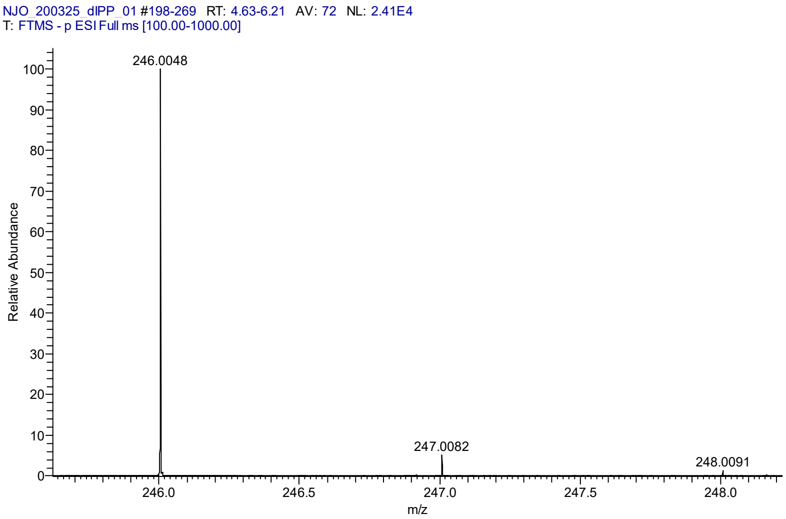

Figure S7. ESI-MS (negative ion) of (*E*)-(4-$^{2}H_{1}$)-IPP.

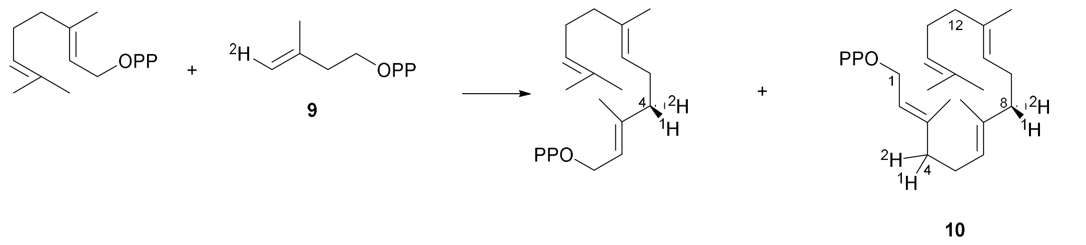

A

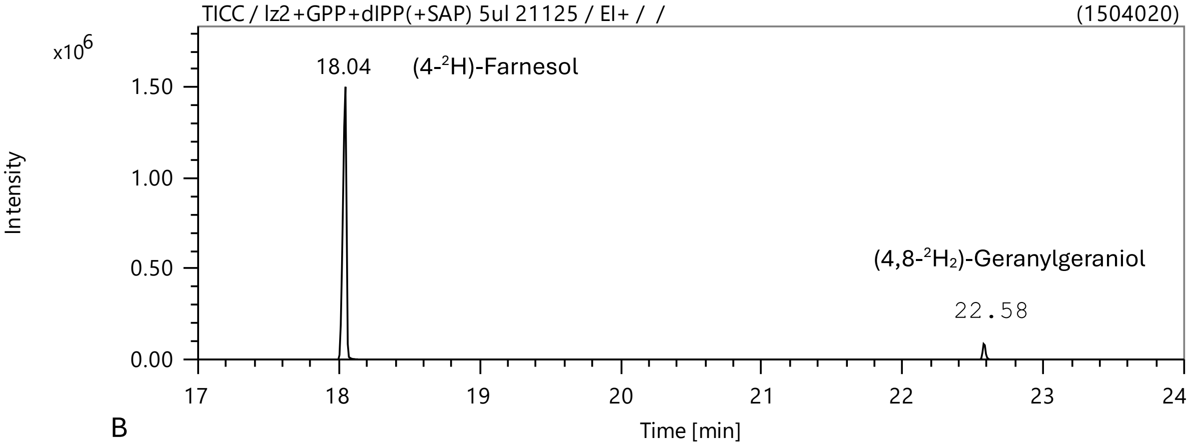

B

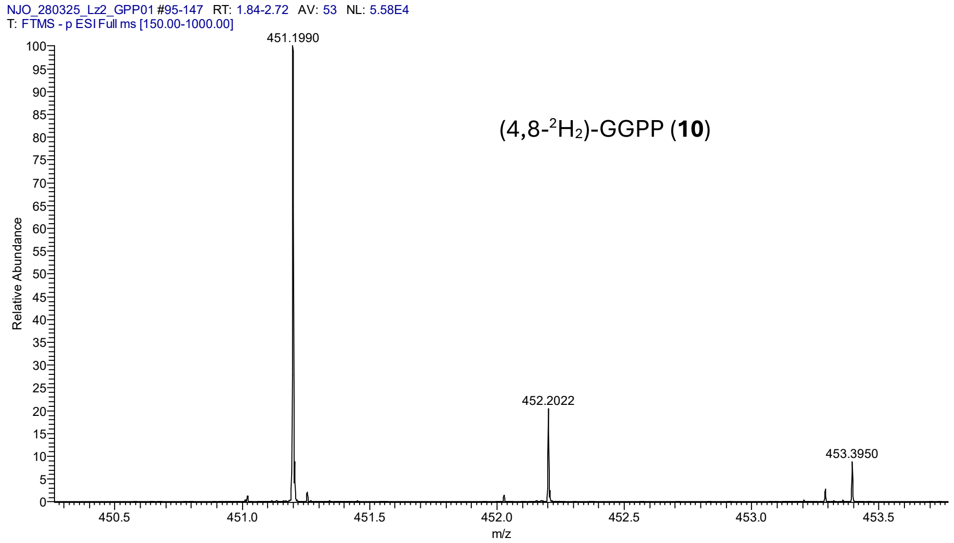

Figure S8. A) GC-MS chromatogram of the products of GPP and deuterated IPP (**9**) catalysed by *Ll*FPPS following hydrolysis by shrimp alkaline phosphatase to yield the alcohols. B) ESI-MS (negative ion) of (4,8-$^2H_2$)-GGPP (**10**) product of GPP and deuterated IPP (**9**) catalysed by *Ll*FPPS. Measured $(M-H)^-$ 451.1990, calculated for $C_{20}H_{33}D_2O_7P_2^-$ 451.1989.

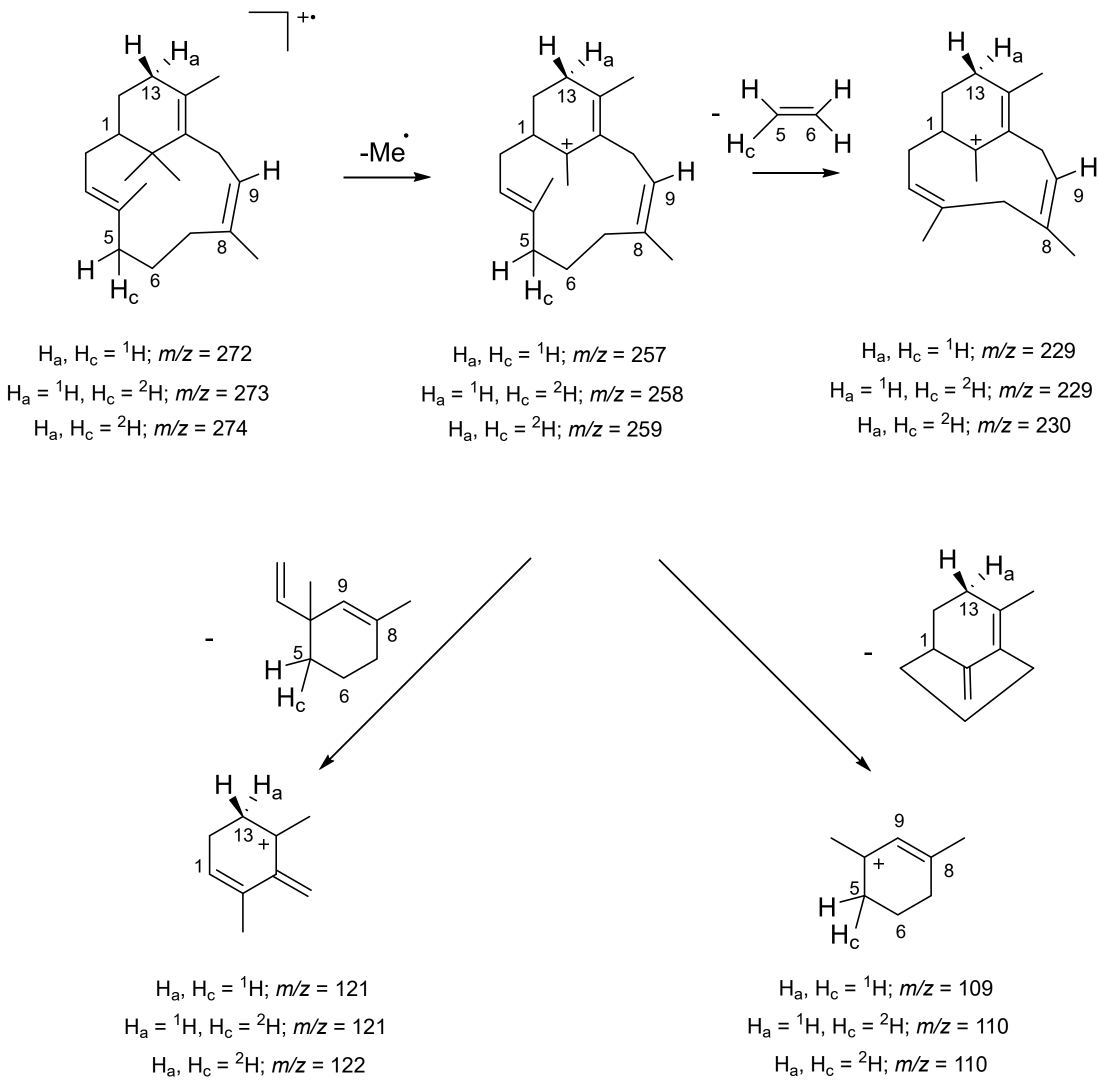

Figure S9. Proposed EI-MS fragmentation of sobralene isotopologues suggesting the origin of some key fragment ions.
